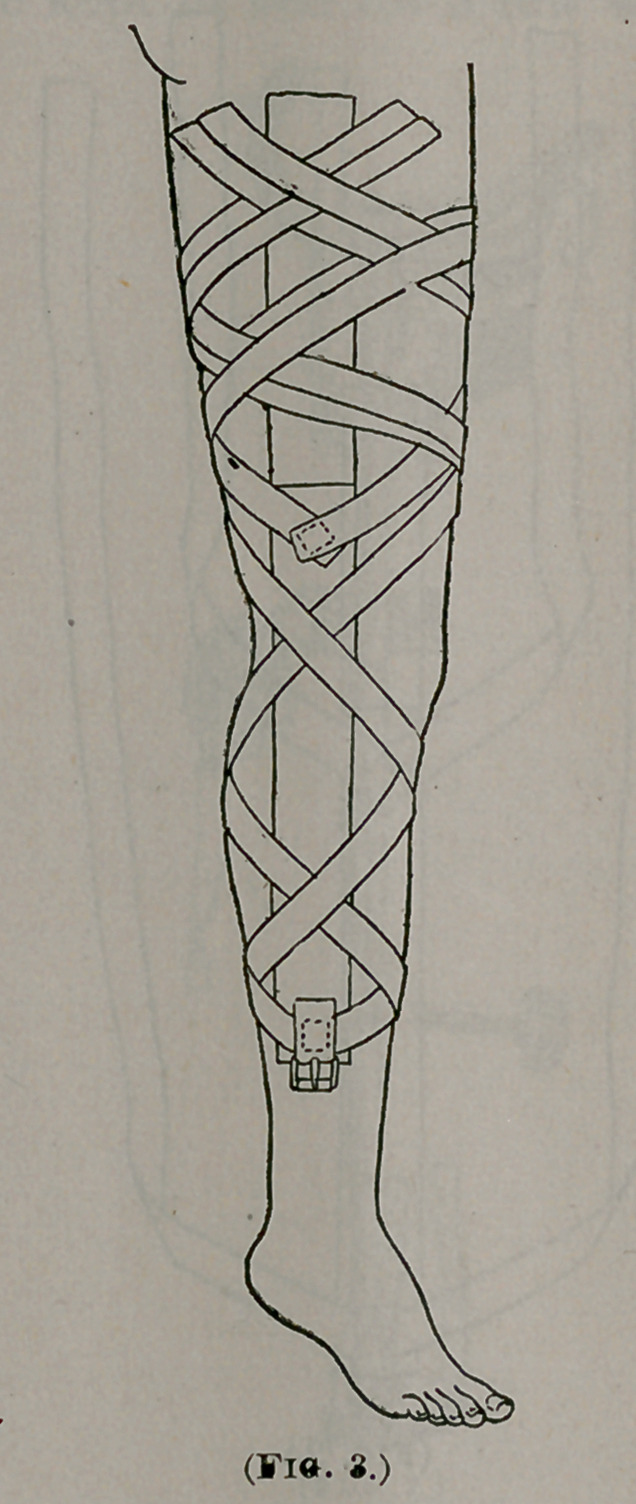# Improved Long Traction Hip-Splint, with Proper Method of Applying Adhesive Plasters*Demonstration before the Surgical Section of the Pan-American Medical Congress, Washington, September 6, 1893.

**Published:** 1893-11

**Authors:** Henry Ling Taylor

**Affiliations:** New York


### Southern Medical Record.

A MONTHLY JOURNAL OF MEDICINE AND SURGERY.

VOL. XXIII. ATLANTA, GA., NOVEMBER, 1893. No. 11.

### Original Articles.

### IMPROVED LONG TRACTION HIP-SPLINT, WITH PROPER METHOD OF APPLYING ADHESIVE PLASTERS.*

BY HENRY LING TAYLOR, M. D., NEW YORK.

In describing an apparatus there is danger that the appliance itself, which is merely a means to an end, should assume undue importance, while the principles, of which the apparatus must be the embodiment, remain in the back-ground.

Even if correct principles are assumed, practical success is always conditioned by the operator's pains-taking attention to the details of treatment, and by his intelligent appreciation of the requirements of different individuals, and of the different phases of the disease. "Intrinsic excellence is of no avail without proper adaptation." The object of the following lines, however, is to describe some of the means employed for securing continuous positive counter-traction for the relief and protection of an inflamed hip-joint, rather than to enumerate the varying indications for treatment, presented by inflammation of the hip-joint in its different stages.

The long traction hip-splint has for its object to allay muscular spasm, to relieve the joint of undue pressure, to prevent

* Demonstration before the Surgical Section of the Pan-American Medical Congress, Washington, September 6, 1893.

undue motion at the hip, to protect the hip-joint from the concussion and strain of the bodily movements, and to take the entire weight of the body when walking is permitted. It is, when properly used, a constantly acting protector to guard the diseased joint from internal and external sources of injury, in order that local irritation and disintegration may be checked, local and general nutrition improved, and deformity prevented or overcome. In order to still better protect the joint, the patient should be kept in bed for a few weeks with a weight of

(FIG. 1.)

six to twelve pounds added to the splint, in the acuter stages of the disease ; but no period of simple weight extension in bed or simple fixation can be made a satisfactory substitute for positive counter-extension in the lines of deformity by a proper apparatus efficiently applied.

Pain, when present, night cries and muscular spasm usually cease in a short time under the latter plan of management, and the patient is soon enabled to move about in comfort, with or without crutches, and to get the benefit of large doses of fresh air, with which nothing can be compared as a blood purifier and tissue regenerator. In other words, a proper splint is simply an aid in the local hygiene of the joint, and the general hygiene of the patient, to be used and modified, in connection with other measures according to the hygienic indications presented.

The improved long traction hip-splint (Fig. 1) consists in a hol-

(FIG. 2.)

low shank firmly secured at its upper end to a side-plate, from which strong curved steel horns spring to carry the perineal strap. In this shank plays a notched bar, which is worked by a key ; this bar is bent under the foot at a right angle and flattened to give attachment to a strap, which is buckled to the adhesive plasters ending just above the malleoli. The improvement in the apparatus described over the original Taylor hip-splint, consists mainly in the substitution of the rigid curved steel horns of peculiar shape, for the horizontal hip-band, long ago discarded in our practice.

This change was made on account of the greater simplicity, convenience and comfort of the new arrangement, but chiefly to give greater rigidity and precision to the apparatus.

The manner of preparing and applying the adhesive plasters is of considerable importance; they should pull evenly and from above the knee. If traction is applied mainly below the

(FIG. 3.)

knee and continued for a considerable length of time, severe injury to the knee may result, of which I have seen deplorable instances. The plasters (Fig. 2) should consist in two strips of diachylon plaster spread on twilled muslin; to the lower end of each plaster a buckle is attached. These strips are one and one-half inches wide, and long enough to reach from just below the level of the groin to two inches above the malleoli. To each strip are sewed two tails of three-quarters of an inch wide rubber plaster above the knee and two tails at the lower end, diverging upward. With the long strips in position, on the outer and inner aspects of the limb respectively, the narrow tails of rubber plaster are to be evenly wound around the limb in a spiral direction upward, beginning with the upper pairs ; while the lower tails are being applied in a similar manner, firm traction should be made from the buckles by an assistant. Plasters applied in this manner (Fig. 3) pull from the thigh, so that the knee is protected, and give a firm basis for continued traction. The plasters are kept in position by a bandage, or better by a laced muslin legging ; they should be inspected from time to time and changed once in six or eight weeks. A useful precaution to further protect the posterior ligament of the knee and add to the patient's comfort is to keep the knee slightly flexed, when the patient is recumbent by placing a folded towel or sheet beneath the popliteal space.

## Figures and Tables

**Fig. 1. f1:**
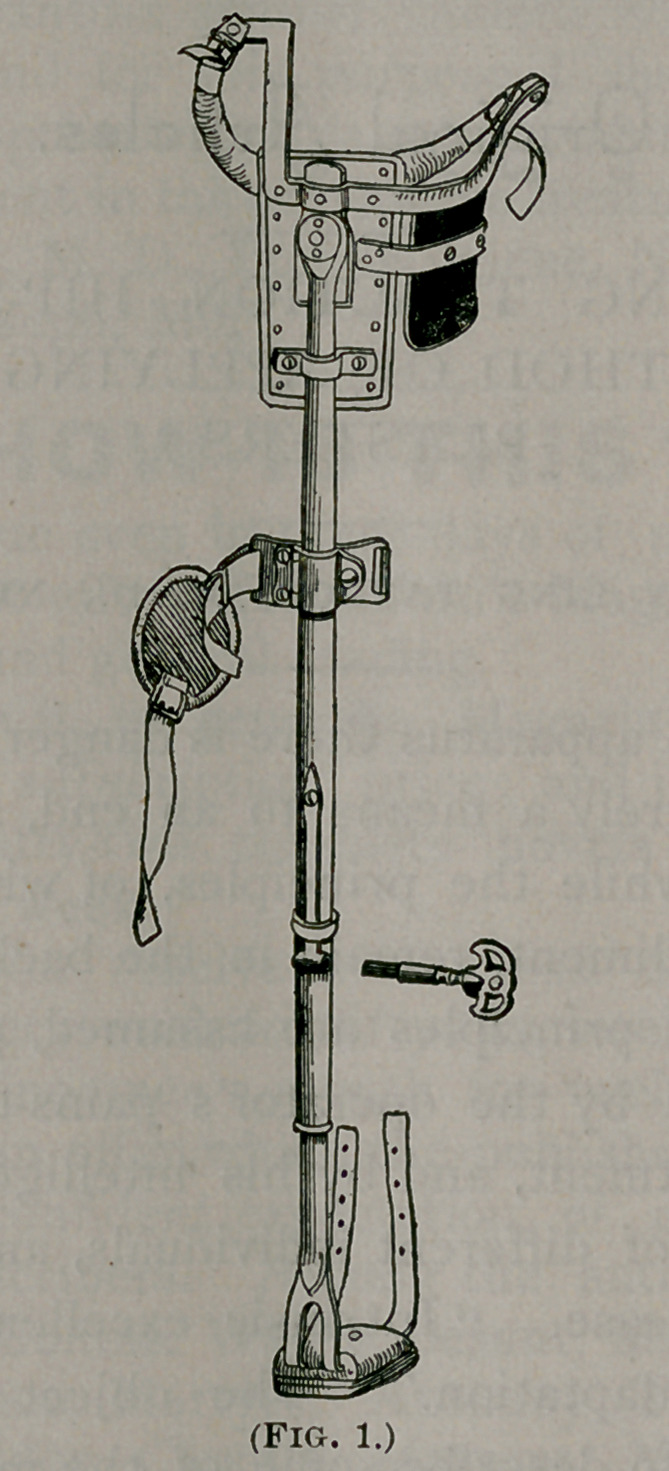


**Fig. 2. f2:**
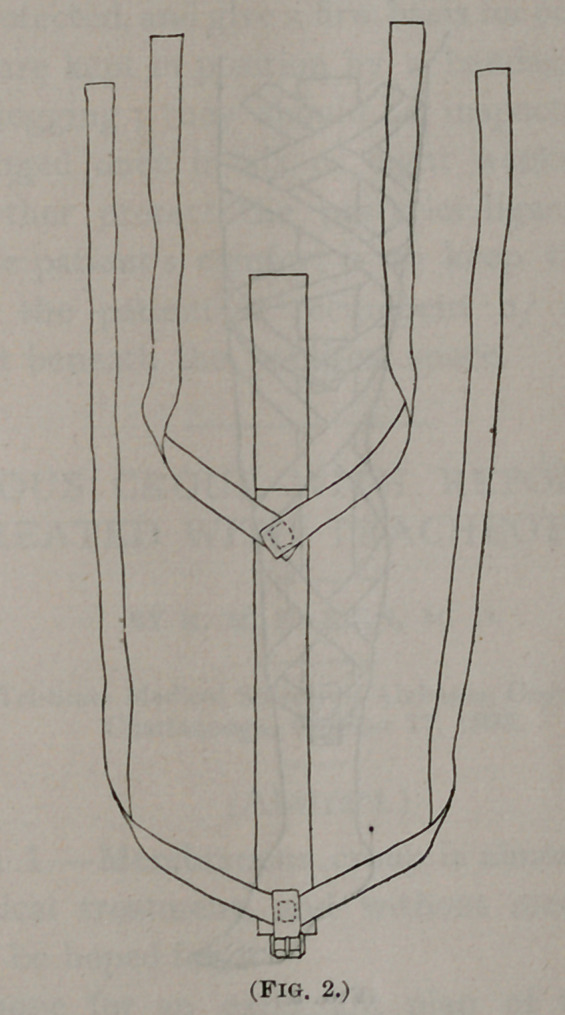


**Fig. 3. f3:**